# A novel laboratory-based nomogram for assessing infection presence risk in acute-on-chronic liver failure patients

**DOI:** 10.1038/s41598-023-44006-9

**Published:** 2023-10-08

**Authors:** Rui Sun, Wenli Lu, Wanhua Ren, Shuhong Zhang, Dongxue Yao, Nannan Zhang, Keqing Zhong, Wenrui Zhao, Xiaolin Tang, Meihong Han, Tao Li

**Affiliations:** 1 Department of Infectious Diseases, Shandong Provincial Hospital, Shandong University, Jinan, China; 2 Department of Infectious Diseases, Shandong Provincial Hospital Affiliated to Shandong First Medical University, 324#, Jing 5 Road, Jinan, China; 3 Department of Infectious Diseases, Jinan Central Hospital Affiliated to Shandong First Medical University, Jinan, China

**Keywords:** Biomarkers, Diseases, Risk factors, Hepatitis, Liver diseases, Infectious-disease diagnostics

## Abstract

Accurate assessment of infection presence risk level, timely diagnosis, and effective control are critical for decreasing mortality of Acute‑on‑chronic liver failure (ACLF). We aimed to develop and validate a novel diagnostic model to accurately assess infection presence risk level in ACLF patients. 185 ACLF patients with/without infection were enrolled, and their demographic, physical findings, immune-inflammatory, hepatic function, metabolism, and coagulation-fibrinolysis indicators were analyzed. Regression analysis was performed to identify the independent diagnostic parameters, which were further used to establish diagnostic models with a nomogram for visual. An area under receiver operating characteristic curve (AUROC), calibration plots, clinical impact curves, decision curve analysis, and net reclassification index were used to evaluate and identify the best model. An external validating cohort was introduced to verify the diagnostic accuracy. We screened out white blood cell (WBC) count, LYM%, blood urea nitrogen (BUN), and D-dimer for assessing infection presence risk levels in ACLF patients. WBD (WBC + BUN + D-dimer) was established and proposed as a novel diagnostic model for infection presence risk levels assessment in ACLF patients with an AUROC of 0.803 (95%CI 0.723–0.883), 0.885 (95%CI 0.786–0.984) in training and external cohorts, respectively. In stratification analysis by ACLF etiology and stages, WBD achieved an AUROC of 0.791 (95%CI 0.691–0.891) and 0.873 (95%CI 0.78–0.966) in HBV-related and early-stage patients, respectively. Whereas a higher AUROC of 0.905 (95%CI 0.807–1.00) in the early-stage of HBV-related ACLF patients indicated its optimum application scope. WBD, a novel laboratory-based nomogram, can serve as a decision-making support tool for clinicians to assess infection presence risk levels in ACLF patients.

## Introduction

Acute-on-chronic liver failure (ACLF) is an acute liver decompensation in chronic liver disease patients, which leads to multi-organ failure and high short-term mortality^1^. The infection has been certified as the main trigger and common complication of ACLF with 50–70% incidence and accounts for the poor outcomes in ACLF patients^2^. Therefore, accurate assessment of the risk level of infection presence, timely diagnosis, and effective control are critical for decreasing mortality and improving the prognosis of ACLF.

During the treatment of ACLF, clinicians often face those challenges: how to judge whether the infection is present now, especially early infection without remarkable physical findings; how to decide whether to perform an invasive and/or expensive examination for further confirmation at once? However, compared with the prediction model for predicting infection within a certain period, the advantage of the diagnostic model is to assess the risk level of infection presence at the cross-sectional time point. Therefore, an accurate diagnostic model can provide clinicians with a useful clinical decision-making support tool to assess the probability of infection presence at the cross-sectional time point, especially during the early stage of treatment.

Currently, several risk factors were shown to be associated with infection development in ACLF patients. It was reported that elevated C-reactive protein (CRP), the presence of advanced hepatic encephalopathy, and elevated white blood cells (WBC) count were independently related to the infection development in ACLF patients^3^, while elevated CRP is also proved as an accurate indicator of bacterial infection presence in autoimmune liver disease-associated ACLF^4^. Recently, Igna et al.^5^ reported that presepsin level ≥ 2300 pg/mL and procalcitonin level ≥ 0.9 ng/mL can be used as non-invasive tools for the early diagnosis of infections presence in ACLF patients. Additionally, a few predictive models based on the immune-inflammatory and hepatic function indexes were also constructed to predict the infection development and prognosis in ACLF with limited predictive efficacy. Zhang Z and Rui et al. reported that two predictive models (one named GIC including serum globulin, IL-6, and CRP; the other model including albumin, CRP, and glucose) can predict the bacterial infection development in HBV-ACLF patients^6,7^. Furthermore, some parameters, such as age, total bilirubin, lactate dehydrogenase, soluble IL-2 receptor, WBC count, etc., were also identified as independent predictors for mortality in ACLF patients with infection^8,9^. However, besides immune-inflammatory responses^10,11^, hypermetabolism and coagulation dysfunction lacking attention have already existed at a very early stage of infection^12,13^. Michael Schwameis et al.^14^ reported that D-dimer, as a traditional indicator of the coagulation-fibrinolysis system, was significantly higher within hours of culture-proven bacteremia and can be a promising marker of lethality already at the onset of infection. Apparently, it is these initial alterations in hypermetabolism and coagulation dysfunction that can be used to develop a diagnostic model for the probability of infection presence assessment in ACLF patients.

In this study, relatively comprehensive detailed indicators associated with demography, hepatic function, hypermetabolism, immune-inflammatory, coagulation dysfunction, and so on were collected from the enrolled ACLF patients with/without infection. Following the rigorous screening of independent diagnostic indicators using regression analysis, we aimed to establish a novel laboratory-based nomogram to assess the infection presence in ACLF patients. In our review, this diagnostic model can help clinicians to make the proper clinical decision at this cross-sectional time point, thus avoiding delayed diagnosis of infection and/or overdiagnosis.

## Results

### Participants characteristics

125 patients with ACLF were finally enrolled in the training cohort, of which 72 (57.6%) were uninfected, and 53 (42.4%) were infected. The external validating cohort included 60 patients with 23 (38.3%) infected and 37 (61.7%) uninfected, matched for age, gender, ACLF etiology, and diabetes (*p* > 0.05 for all). Infected patients had a higher frequency of ascites, WBC counts, Neutrophil (NEU) %, NEU counts, Monocyte (MON) counts, Indirect Bilirubin (IBIL), Blood urea nitrogen (BUN), Creatinine (CRE) and D-dimer level compared to uninfected patients. Other parameters that had significant differences between uninfected and infected patients are shown in Table 1 and Figure S1. These results provided a feasible program for screening the valuable diagnostic parameters to evaluate the risk level of infection presence in ACLF patients.

**Table 1:** Baseline characteristics of ACLF patients in the training cohort.

Variables (n = 31)	Uninfected patients (n = 72)	Infected patients (n = 53)	*p*
Age (IQR) (years)	47.50 (42.00, 53.50)	48.00 (39.00, 56.00)	0.928
Gender			
Male, n (%)	58 (80.6)	41 (77.4)	0.663
Female, n (%)	14 (19.4)	12 (22.6)	
Body temperature (IQR) (℃)	36.50 (36.20, 36.82)	36.30 (36.20, 36.80)	0.16
Heart beats (IQR) (Bpm)	88.00 (76.00, 95.25)	86.00 (76.00, 94.00)	0.778
Respiratory rate (IQR) (Rpm)	20.00 (18.00, 22.00)	20.00 (18.00, 21.00)	0.672
Alcohol abuse, n (%)			
Present	21 (29.2)	16 (30.2)	1
Absent	51 (70.8)	37 (69.8)	
HBV infection, n (%)			
Present	56 (77.8)	38 (71.7)	0.53
Absent	16 (22.2)	15 (28.3)	
ALD, n (%)			
Present	9 (12.5)	7 (13.2)	1
Absent	63 (87.5)	46 (86.8)	
Diabetes, n (%)			
Present	12 ( 16.7)	6 ( 11.3)	0.45
Absent	60 ( 83.3)	47 ( 88.7)	
Ascites, n (%)			
Present	49 (68.1)	48 (90.6)	0.004*
Absent	23 (31.9)	5 (9.4)	
WBC (IQR) (10^9^/L)	5.60 (3.92, 8.22)	9.24 (6.07, 13.16)	< 0.001*
RBC (IQR) (10^12^/L)	4.07 (3.38, 4.57)	3.71 (3.27, 4.28)	0.139
HB (IQR)(g/L)	127.00 (111.00, 143.25)	124.00 (109.00, 136.00)	0.313
PLT (IQR)(10^9^/L)	88.10 (67.50, 114.50)	92.00 (58.00, 157.00)	0.541
LYM% (IQR)	23.70 (15.57, 32.00)	14.30 (8.10, 22.40)	< 0.001*
MON% (SD)	10.17 (3.73)	8.91 (4.46)	0.086
NEU% (IQR)	65.55 (56.35, 72.85)	74.80 (65.10, 84.50)	< 0.001*
LYM (IQR)(10^9^/L)	1.34 (0.98, 1.64)	1.12 (0.79, 1.65)	0.214
MON (IQR)(10^9^/L)	0.59 (0.40, 0.75)	0.82 (0.43, 1.22)	0.001*
NEU (IQR)(10^9^/L)	3.37 (2.30, 5.47)	6.07 (4.10, 10.70)	< 0.001*
PCT (IQR)(ng/ml)	0.51 (0.35, 0.64)	0.60 (0.46, 0.77)	0.249
CRP (IQR)(ng/ml)	10.94 (7.21, 15.70)	13.59 (9.05, 20.55)	0.071
AST (IQR)(U/L)	170.50 (89.75, 398.00)	161.00 (84.00, 381.00)	0.508
ALT (IQR)(U/L)	207.00 (63.50, 512.75)	120.00 (58.00, 256.00)	0.096
GGT (IQR)(U/L)	108.50 (66.50, 149.25)	81.00 (44.00, 141.00)	0.111
ALP (IQR)(U/L)	183.00 (153.00, 220.25)	153.00 (118.00, 221.00)	0.029*
ALB (IQR)(g/L)	30.05 (27.58, 34.00)	29.30 (25.80, 32.00)	0.052
TBIL (IQR)(μmol/L)	307.15 (210.30, 413.70)	349.48 (214.90, 401.70)	0.221
DBIL (IQR)(μmol/L)	182.05 (113.44, 297.70)	218.00 (119.43, 286.00)	0.421
IBIL (IQR)(μmol l/L)	102.20 (85.76, 126.60)	119.10 (92.90, 154.79)	0.042*
BUN (IQR)(mmol/L)	3.90 (3.30, 5.93)	7.00 (4.70, 11.20)	< 0.001*
CRE (IQR)(μmol L)	62.15 (52.95, 76.25)	69.00 (57.00, 94.00)	0.045*
D-dimer (IQR)(mg/L)	1.95 (0.66, 3.30)	3.62 (1.83, 5.55)	< 0.001*
PT (IQR)(s)	21.20 (18.35, 26.12)	21.80 (18.00, 27.40)	0.912
INR (IQR)	1.85 (1.58, 2.28)	1.94 (1.51, 2.39)	0.96

*p < 0.05 for significance.

### Candidate predictors for infection presence from the training set

After using the Tolerance and the Variance inflation factor (VIF) to analyze the multicollinearity between variables, we found there was severe multicollinearity among variables of the training cohort (Table. S1, Fig. S2a). Thus, Lasso regression analysis was performed to identify the potential risk factors of infection in ACLF patients. Among these 11 candidate variables, WBC count, LYM%, BUN, and D-dimer were eventually identified as potential diagnostic parameters for infection presence in ACLF patients and were with nonzero coefficients in the Lasso regression analysis (Table. 2, Fig. S2b,c). Then, multivariate regression analysis was performed to identify the candidate diagnostic parameters. WBC count (OR 1.187, 95%CI 1.047–1.378; *p* = 0.015), BUN (OR 1.105, 95%CI 0.988–1.256; *p* = 0.0896), D-dimer (OR 1.271, 95%CI 1.037–1.5780; *p* = 0.024) were finally identified as independent diagnostic parameters for the infection presence in ACLF patients. Despite the marginal significance of BUN (OR 1.105, 95%CI 0.988–1.256; *p* = 0.0896), it was also enrolled in the model and did not affect the predictive ability of the model (Table.2).

**Table 2:** Lasso regression and multivariate logistic analysis of predictors for the infection presence in ACLF patients in the training cohort.

Variables	Lasso analysis	Multivariate analysis		
	Regression coefficient	OR	95%CI	*P*
WBC count (10^9^/L)	0.021	1.187	(1.047,1.378)	0.015*
LYM%	− 0.013	0.984	(0.928,1.041)	0.573
BUN (mmol/L)	0.0196	1.105	(0.988,1.256)	0.0896^†^
D-dimer (mg/L)	0.011	1.271	(1.037,1.580)	0.024*

*p < 0.05 for significance.

^†^*p* < 0.1 for marginal significance.

In order to verify the diagnostic accuracy of parameters for infection in ACLF, ROC analysis of WBC count, LYM%, BUN, and D-dimer was plotted with an area under the receiver operating characteristic curve (AUROC) (specificity, sensitivity, 95% CI) of 0.746 (69%, 74%, 0.656–0.835), 0.728 (78%, 58%, 0.637–0.819), 0.733 (58%, 83%, 0.7638–0.827), 0.693 (42%, 91%, 0.600–0.786), respectively. Meanwhile, the correlations between these independent diagnostic parameters revealed WBC count was positively correlated with BUN (*R* = 0.428, *p* < 0.0001), and BUN was positively correlated with D-dimer (*R* = 0.446, *p* < 0.0001) for validating the feasibility of their combination to develop the diagnostic model (Fig. S3).

### The novel laboratory-based model development and validation

**Figure 1:**
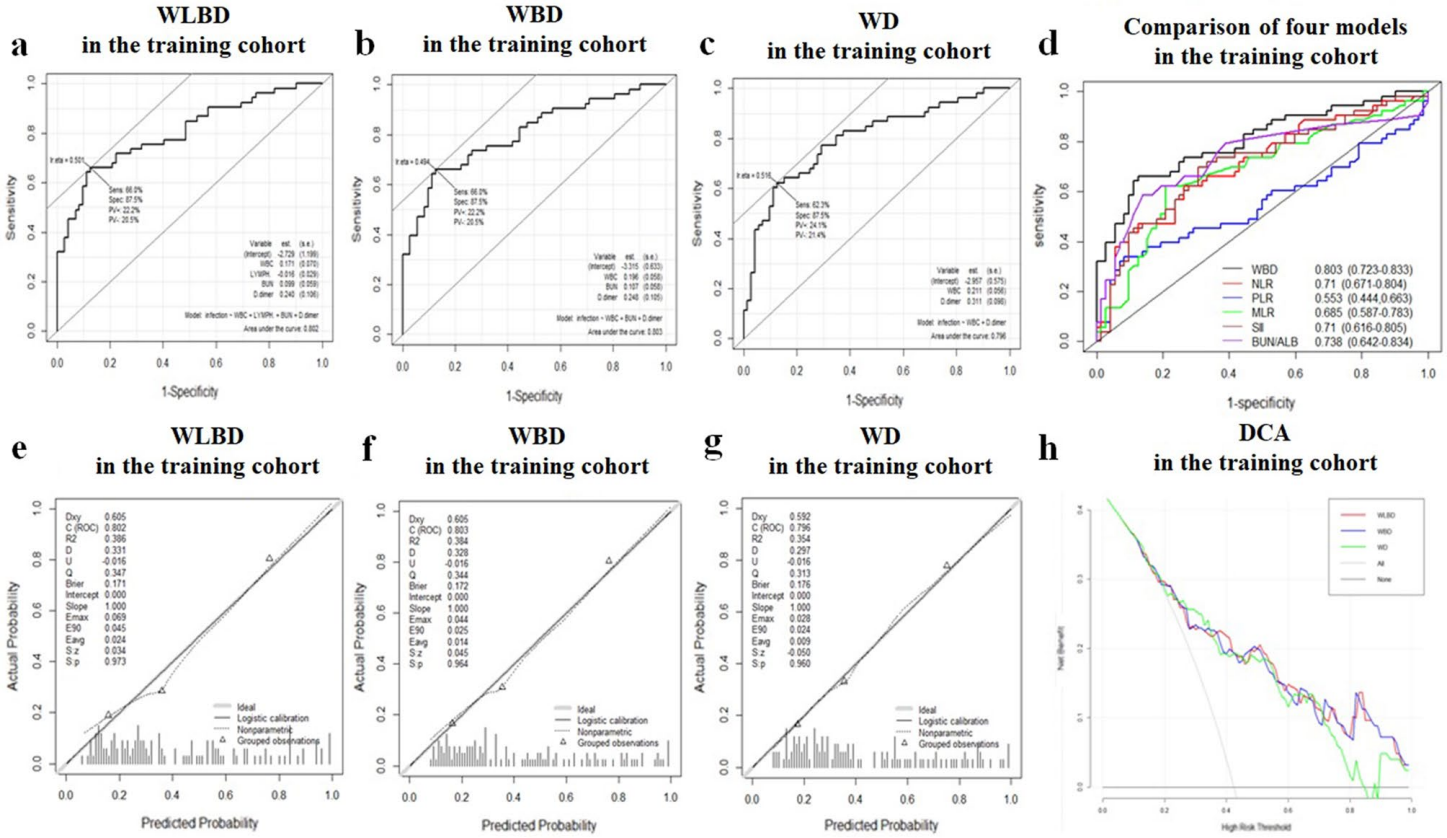
Receiver operating characteristic curve (ROC), Calibration plots, and decision curve analysis (DCA) of 3 diagnostic models in the training cohorts. The ROC curves of the WLBD (**a**), WBD (**b**), and WD (**c**) were plotted to verify the diagnostic accuracy of these models in training cohort. The ROC curves of other 5 various diagnostic models related to infection were performed to be compared with that of WBD in the training cohort (**d**). The calibration plot of WLBD (**e**), WBD (**f**), and WD (**g**) was plotted to verify the consistency of the model in the training cohort. The DCA curves of WBLD, WBD, and WD were plotted to verify the net benefit of these models in the training cohort (**h**).

For improving diagnostic accuracy, WBC count, LYM%, BUN, and D-dimer were randomly combined to develop three fitted and simplified predictive models named as follows: WLBD (WBC count, LYM%, BUN, D-dimer), WBD (WBC count, BUN, D-dimer), and WD (WBC count, D-dimer). The AUROC (specificity, sensitivity, 95%CI) of these models were 0.802 (87.5%, 66%, 0.722–0.882), 0.803 (87.5%, 66%, 0.723–0.883), and 0.796 (87.5%, 62.3%, 0.714–0.877) (Fig. 1a,b and c), respectively. In addition, they all have good calibration performance validated by the Hosmer–Lemeshow test (*p* = 0.531; *p* = 0.7205; *p* = 0.8121). What’s more, the diagnostic accuracy of some common immune-inflammation indexes, such as neutrophile-to-lymphocyte ratio (NLR), platelet-to-lymphocyte ratio (PLR), monocyte-to-lymphocyte ratio (MLR), systemic immune-inflammation index (SII) and BUN-to-albumin ratio (BUN/ALB)^15–17^, were also evaluated in this study, but none of them exhibited better diagnostic accuracy than our proposed WBD model (Fig. 1d).

**Figure 2:**
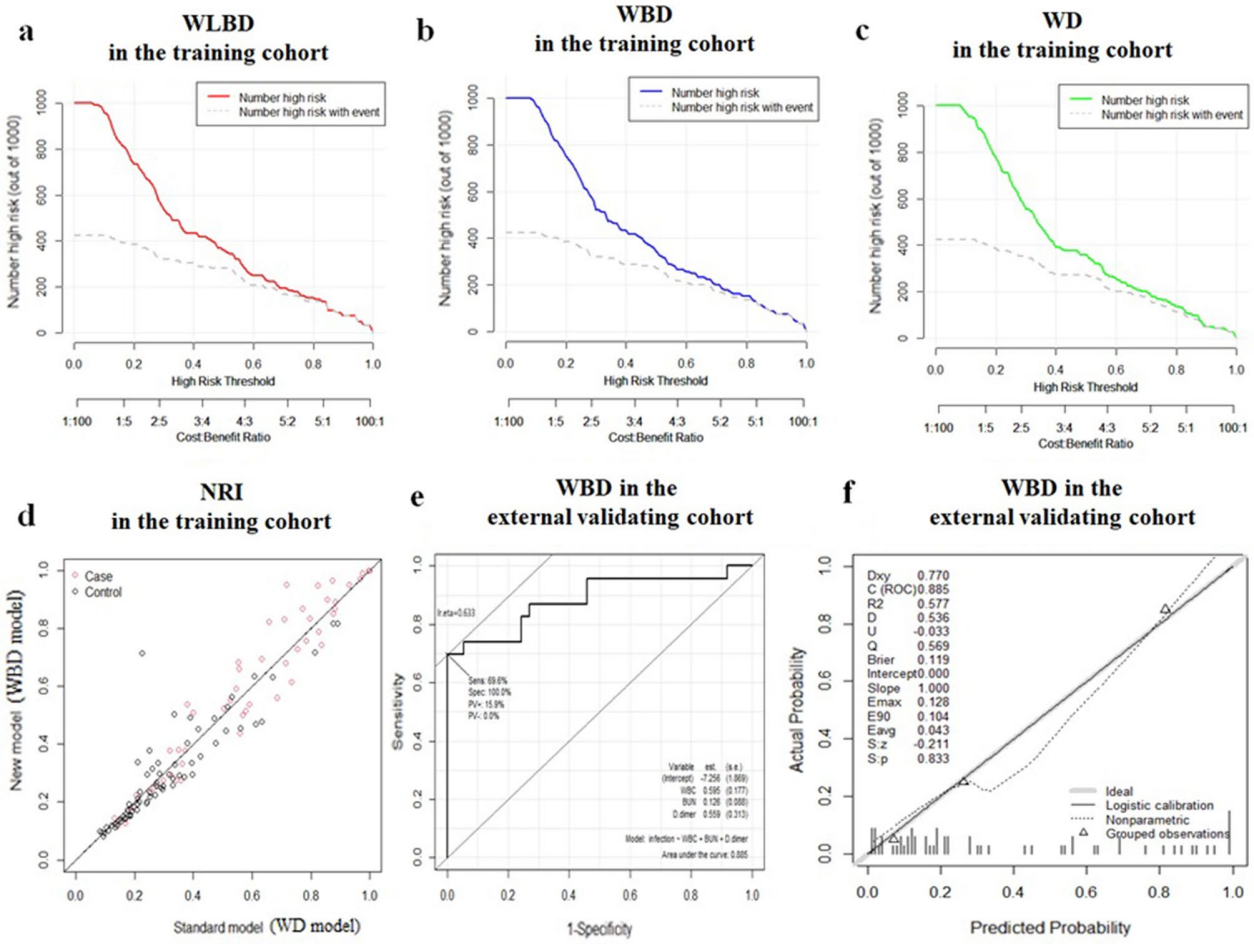
The clinical impact curves and net reclassification improvement (NRI) and Receiver operating characteristic curve (ROC) of the diagnostic models in the training and external validating cohort. The clinical impact curves of the WLBD (**a**), WBD (**b**), and WD (**c**) were plotted to verify the clinical utility of these models in the training cohort. The NRI of WD between WBD (**d**) was calculated to identify the optimization model. The ROC curve (**e**) and calibration plots (**f**) of WBD model were plotted in the external validating cohort.

To confirm the consistency and clinical utility of these models, the calibration plots, decision curve analysis (DCA), and clinical impact curves were plotted. The calibration plot for the probability of models showed good consistency between the prediction and actual observation (Fig. 1e,f, and g). The DCA plots and the clinical impact curves indicated that these models presented a great net benefit with a wide range of threshold probabilities for assessing infection presence (Figs. 1h, 2a, b, and c).

Determining which model exhibited superior diagnostic accuracy was the next issue of concern. However, compared with the other two models, WLBD not only included more variables, but also demonstrated no advantage in terms of diagnostic accuracy and calibration performance. Moreover, the net reclassification index (NRI) evaluated whether adding BUN could improve the efficiency of the standard model WD. The NRI for adding BUN from the WD model was 0.436 (95%CI 0.021–0.824 *p* = 0.035), revealing the accuracy of prediction by the new model added BUN (WBD model) was better than that of the traditional model (Fig. 2d).

According to the results, WBD with higher diagnostic accuracy and fewer parameters was finally proposed as a novel diagnostic model for evaluating the risk level of infection presence in ACLF patients. The sensitivity and specificity of the WBD model were 66% and 87.5%, respectively. Meantime, for the WBD model, similar results were also obtained from the external validating cohort with the AUROC of 0.885 (Fig. 2e) and Calibration plot (Fig. 2f). Then, WBD was evaluated with the Hosmer–Lemeshow test, which showed great calibration performance in the external validating cohort (WBD: *p* = 0.0614).

### Construction of the WBD

**Figure 3:**
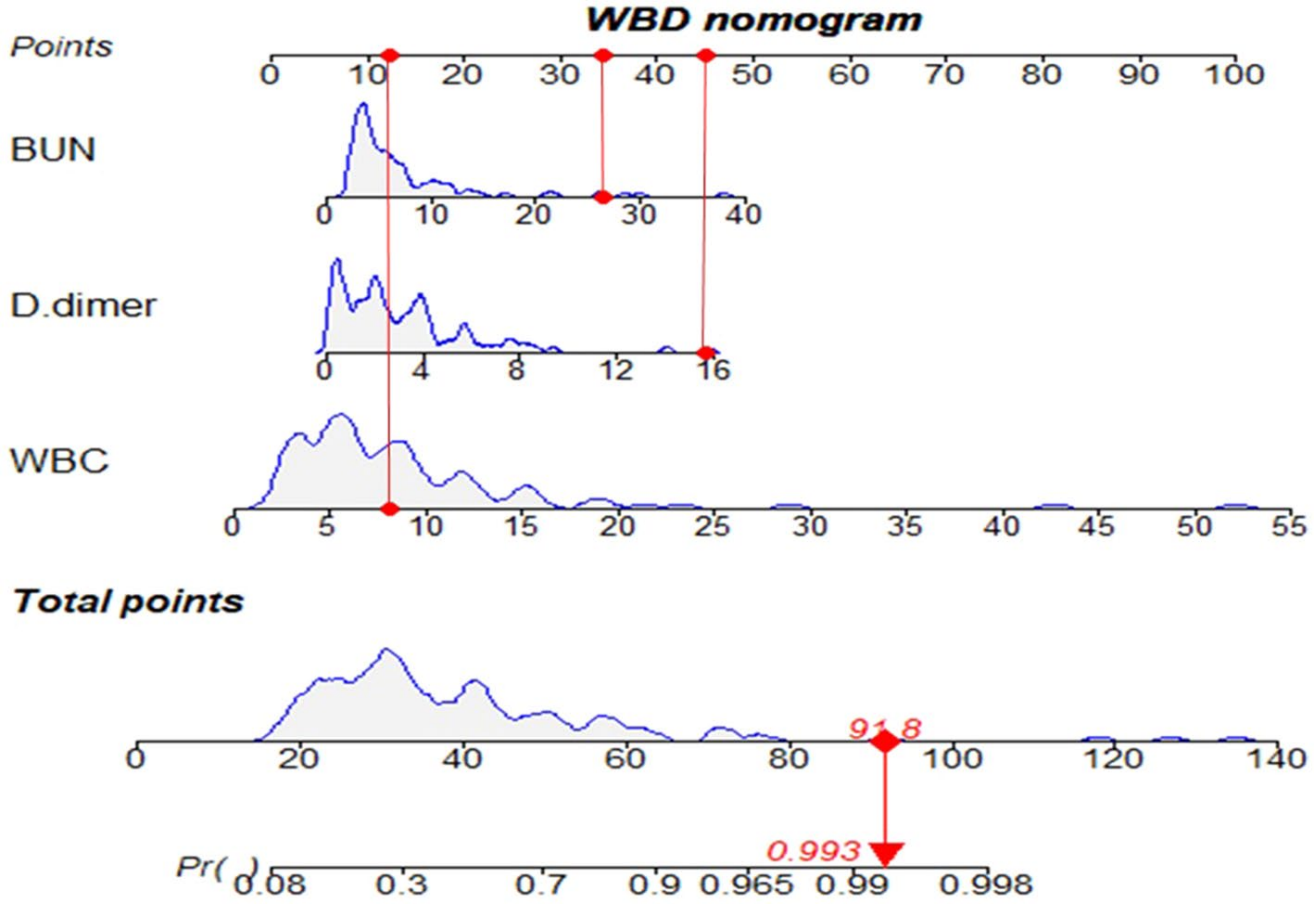
The novel laboratory-based nomogram: WBD.

A nomogram of WBD was developed to visualize and instruct the model more specifically. As an example to better illustrate how to use the nomogram model, consider an ACLF patient with a WBC count of 8.13 × 10^9^/L, BUN of 26.4 mmol/L, and D-dimer level of 15.69 mg/L, represented by the red dot on each line in the nomogram. The total point is 91.8, corresponding to the probability of infection presence in this ACLF patient is estimated to be 0.993 (Fig. 3).

To further validate the diagnostic accuracy of the WBD nomogram in the training cohort, WBD was measured by the fivefold cross-validation technique. The mean AUCROC value of five cross-validation splits was 0.780 suggesting the WBD nomogram had good diagnostic accuracy (Fig. S4).

### Stratification analysis of WBD

**Figure 4:**
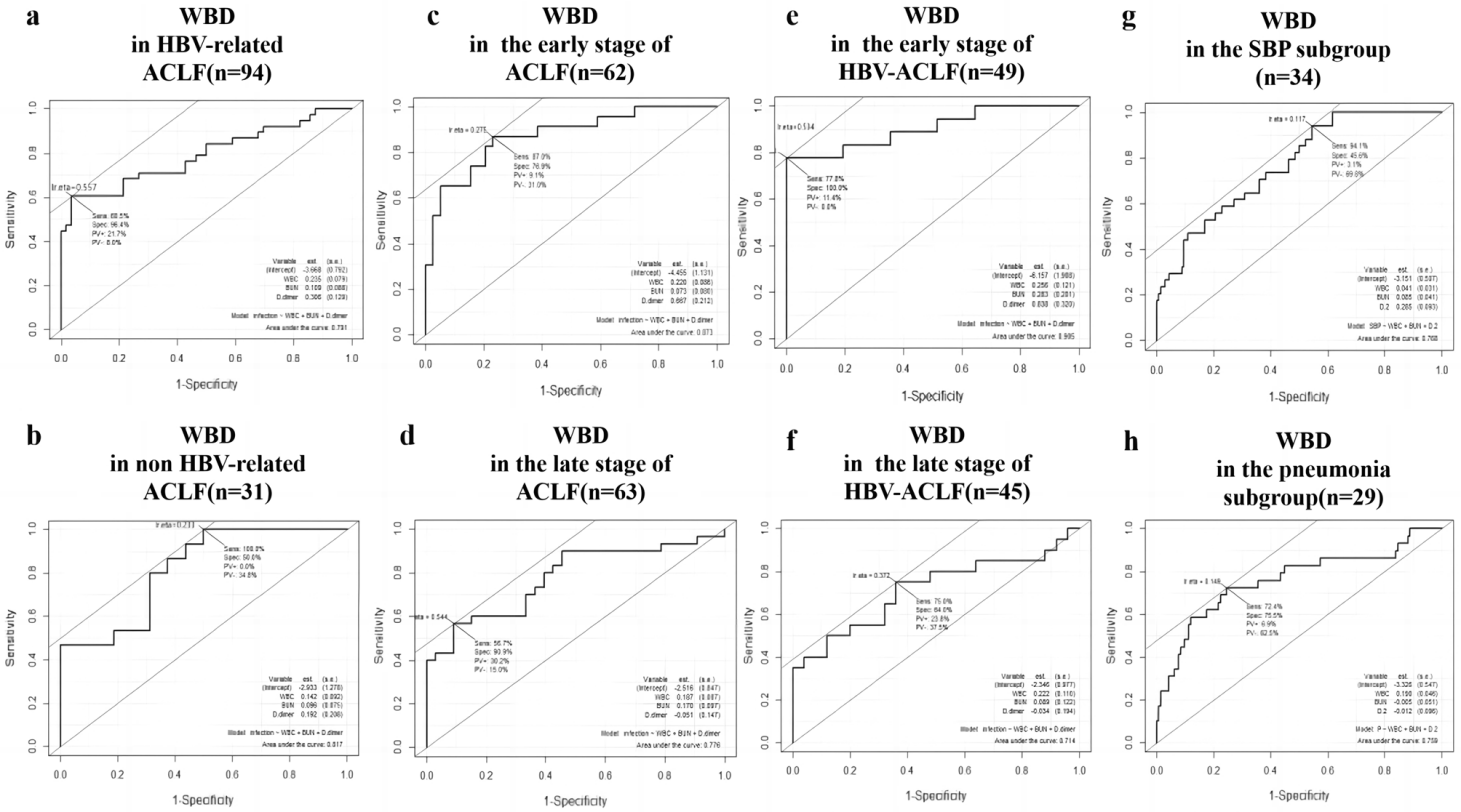
Receiver operating characteristic curve (ROC) of WBD for assessment of the risk level of infection presence in different ACLF etiology and stages. ROC curves of the WBD nomogram in HBV-related ACLF patients’ subgroup (n = 94) (**a**), in non HBV-related ACLF patients subgroup (n = 31) (**b**), in the early-stage of ACLF patients subgroup (n = 62) (**c**), in the late-stage of ACLF patients subgroup (n = 63) (**d**), in the early-stage of HBV-related ACLF patents subgroup (n = 49) (**e**), in the late-stage of HBV-related ACLF patients subgroup (n = 45) (**f**), in the SBP subgroup (n = 34) (**g**), and in the pneumonia subgroup (n = 29) (**h**).

To optimize the clinical application of the WBD nomogram, we applied stratification analysis with different ACLF etiology and stages. As shown in Fig. 4a,b, we divided the patients into 2 subgroups by ACLF etiology as follows: (1) hepatitis B virus (HBV)-related ACLF (n = 94); (2) non-HBV-related ACLF (n = 31). The AUROC of WBD in these two subgroups were 0.791 (95%CI 0.691–0.891), and 0.817(95%CI 0.668–0.966), respectively. In addition, ACLF stages were also stratified into two groups by 2018 Guidelines for the diagnosis and treatment of liver failure in China^1^ as follows: early-stage (prophase and early stage, n = 62) and late-stage (middle and end stage, n = 63) subgroups with AUROC 0.873 (95%CI 0.78–0.966), 0.776 (95%CI 0.657–0.895), respectively (Fig. 4c,d). What’s more, WBD can achieve an AUROC of 0.905 (95%CI 0.807–1.00) in HBV-related ACLF patients in the early-stage (prophase and early stage) subgroup (Fig. 4e), which indicate the optimum application scope for WBD, whereas AUROC of WBD was only 0.714 (95%CI 0.552–0.876) in the late-stage subgroup (Fig. 4f).

In this study, the main types of infection in ACLF patients enrolled in the training cohort were SBP (n = 23, accounted for 43.4%), and pneumonia (n = 17, accounted for 32.1%), which are in concordance with the main types in the validation cohort (SBP: n = 11, accounted for 47.8%, pneumonia: n = 12 accounted for 52.2%) (Table. S2). The AUROC of WBD for assessing the risk level of SBP and pneumonia were 0.768 (95%CI 0.685–0.851), 0.759 (95%CI 0.648–0.870), respectively (Fig. 4g,h). However, when WBD was applied to assess the risk of both types of infections, the AUROC can achiveved 0.825 (95%CI 0.797–0.894).

## Discussion

In this study, WBD was developed and can assess the infection presence risk level in ACLF patients with high accuracy (AUROC:0.803 in the training cohort; AUROC: 0.885 in the external validation cohort). WBD can help clinicians to make the proper clinical decision to avoid the delayed diagnosis of infection and/or overdiagnosis at the cross-sectional time point, especially in early treatment.

It has been reported that hypermetabolism and coagulation dysfunction exist at the onset of infection^12^. Hypermetabolism can induce negative nitrogen balance and eventually increase nitrogen excretion^18^. Additionally, recent advances in the pathogenesis of infection revealed that infection can cause a coagulation dysfunction, mainly for hypercoagulable and hyperfibrinolysis states in the initial phase of infection^13^. Hence, these diagnostic models based on the corresponding indexes derived from hypermetabolism and coagulation dysfunction are promising to offer lots of potential advantages in the accurate evaluation of infection presence risk level. In our study, using the parameters from the immune-inflammatory, hypermetabolic, coagulation dysfunction, and hepatic function, we screened out WBC counts, BUN and D-dimer to evaluate the infection presence risk level in ACLF.

Physical findings, such as fever, elevated respiratory rate, and tachycardia are associated with poor prognosis in ACLF with severe infection and sepsis^19,20^. In this study, no significant differences were observed between the infected and uninfected patients. This may be because the infected patients enrolled were mostly in the early phase and showed no remarkable physical findings. Additionally, the main types of infection were spontaneous peritonitis (n = 23, accounted for 43.4%), and pneumonia (n = 17, accounted for 32.1%). Instead of fever, elevated respiratory rate, and tachycardia, the clinical presentation with abdominal and respiratory symptoms, such as abdominal pain, cough, and expectoration instead of fever, elevated respiratory rate, and tachycardia., were more frequently observed in these two types of infected patients, respectively.

BUN is a biomarker of nitrogen excretion caused by hypermetabolism^21^ and is often elevated in pneumonia, bacteremia, genitourinary tract infections, and sepsis^17,22–24^. In this study, BUN was elevated and identified as an independent diagnostic parameter for infection presence in ACLF (AUROC:0.733; OR:1.105), consistent with previous research^25^. It can be interpreted that elevated BUN, which reflects a net negative nitrogen balance, comes from protein catabolism acceleration because of the sustained potential hypermetabolism induced by infection^12,26,27^. Actually, it has been reported that BUN elevation in ACLF patients is associated with severe infection and renal insufficiency, resulting from reduced BUN extraction in urine^28,29^. However, the mechanism of BUN elevation in our study is completely different. This elevation of BUN was not due to renal insufficiency since all patients had normal CRE levels (see detail in Table 1).

We also identified D-dimer as a key diagnostic parameter for infection presence in ACLF patients. Infection can directly damage the vascular endothelial cells and expose the basement membrane. The latter can recruit tissue factors, activate the coagulation-fibrinolysis system, and result in a hypercoagulable and hyperfibrinolysis state ultimately^30–32^. D-dimer, the fibrinolytic degradation product of cross-linked fibrin, is an ideal biomarker for coagulation dysfunction^33,34^, and elevated levels are observed in various infection conditions, including bacteremia and sepsis^14,35^. EI Gohary et al.^36^ also reported that D-dimer showed good performance in the diagnosis of SBP in patients with liver cirrhosis. Meanwhile elevated D-dimer levels are associated with liver failure. The mechanism is that systemic inflammation, playing a crucial role in the development of ACLF, is well known to be accompanied by the activation of the coagulation-fibrinolysis system. The secondary fibrinolysis caused by intrahepatic hypercoagulability induces elevated D-dimer level^37^. Furthermore, the decreased synthetic capacity of the liver reduces the synthesis of anti-fibrinolytic enzymes and other enzymes, which also makes a hyperfibrinolysis state with elevated D-dimer. Consistent with these studies, our study also shows that the D-dimer has good diagnostic accuracy for infection presence in ACLF patients (AUROC: 0.693; OR: 1.271). These complex relationships between ACLF, infection, and the coagulation-fibrinolysis system offer new evidence for our results. To our knowledge, both infection and coagulation-fibrinolytic dysfunction can be induced by ACLF, and the elevation of D-dimer caused by hyperfibrinolysis has already been reported as a risk factor for infection. Therefore, when the D-dimer level is elevated in ACLF patients, it may suggest they are at a higher infection presence risk level.

As a typical immune-inflammatory reaction indicator, WBC count was also screened out. It was reported that WBC count was more elevated in ACLF infected patients^2,3,25^. The elevated WBC count can not only be induced by infection but also by ACLF itself. The mechanism underlying systemic inflammation and immune system dysfunction in the development of ACLF and infection is complex^38,39^. Furthermore, WBC count is also an important stress response^40^. The infection, as a common stressor, can also cause the elevation of WBC count. Similar to these researches, WBC count was found to be elevated in ACLF infected patients and identified as an independent diagnostic parameter for infection presence in this study (AUROC: 0.746; OR: 1.187).

**Figure 5:**
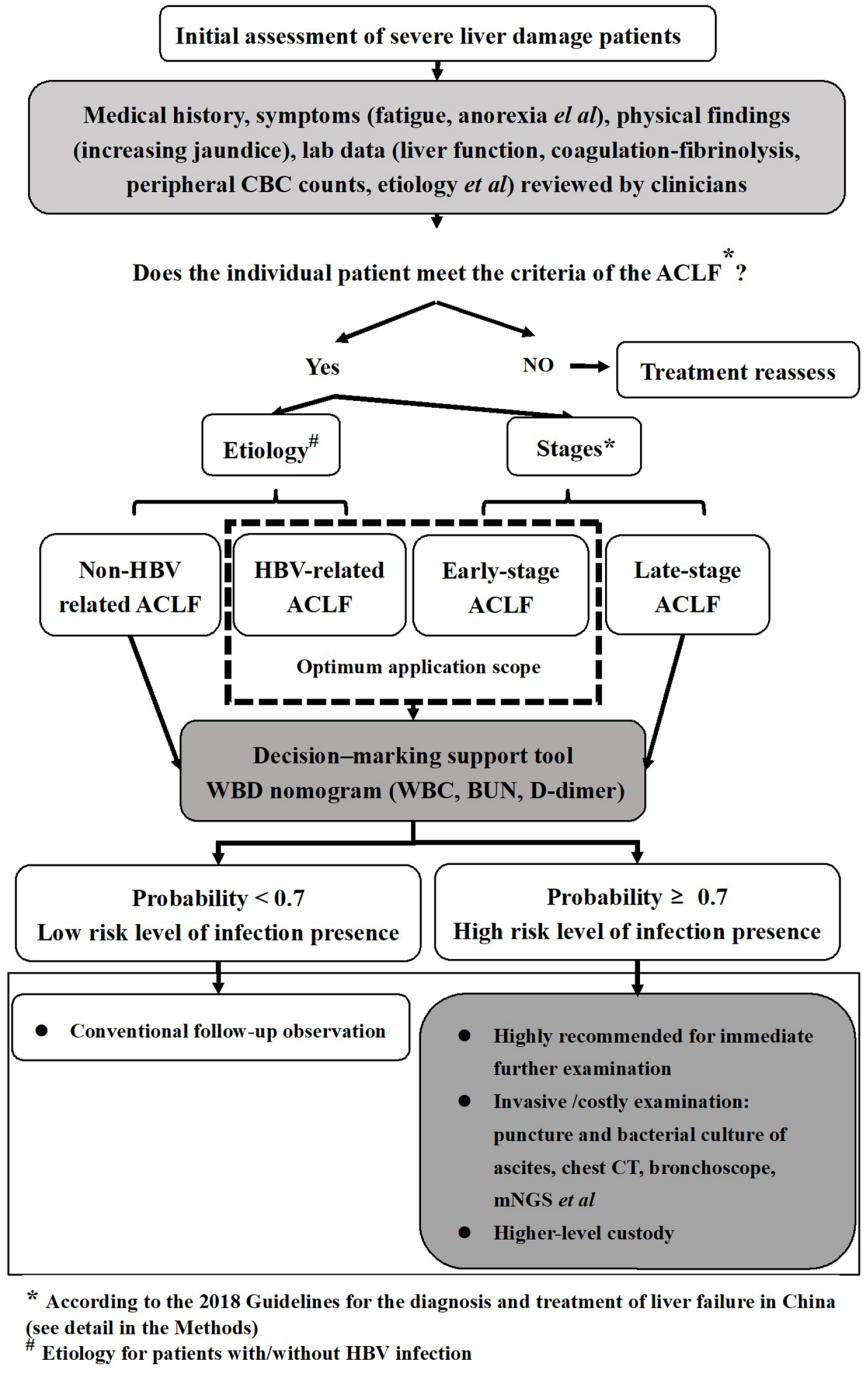
The guideline of the application of WBD.

With routine intravenous blood sample collection, rapid measurement, and accurate laboratory results, WBC counts, BUN, and D-dimer level measurements are commonly available. According to the WBD nomogram, clinicians can gather the necessary indexes in ACLF patients, and calculate the final score corresponding to the infection presence risk in ACLF patients. Here the guideline for the application of WBD can be suggested in Fig. 5: (i) The clinicians diagnose ACLF according to the diagnostic criteria; WBC count, BUN, and D-dimer level were also measured at the time of admission; (ii) as shown in Fig. 4, the total points were calculated by summing the points identified on the “Point” scale for each predictor. By comparing the “Total points” scale and the “Risk” scale, the individual risk level of infection presence could be obtained; (iii) if the probability is above 0.7, the patients will be classified as “high-risk level of infection presence” and highly recommended for further examination, including invasive and/or costly examination, to determine its type and presence. Otherwise, they can continue to receive conventional treatment and follow-up observation. WBD provides clinicians with a convenient clinical decision-making support tool for assessing the risk level of infection presence in ACLF patients, thus avoiding both delayed diagnosis and overdiagnosis.

There are indeed some differences in the diagnostic criteria for acute-on-chronic liver failure (ACLF) from various associations including European Association for the Study of the Liver (EASL), Asian Pacific Association for the Study of the Liver (APASL), American College of Gastroenterology (ACG) and Chinese Medical Association (CMA) et al. The significant reasons for these divergences in criteria for ACLF are attributed to the variations in the ethnicities and the differences in etiological factors for the ACLF. For instance, unlike Europe and North America, where alcohol is the major etiology for ACLF, HBV-related ACLF is more common in the Asia–Pacific regions^41^. Actually, there are many significant differences in the pathological process and clinical characteristics between these two types. HBV-related ACLF commonly experiences liver and coagulation failures, while non-HBV-related ACLF patients more frequently experience kidney and cerebra failure^42,43^. The diagnostic accuracy of WBD in different ACLF etiology and stages were also evaluated in this study. WBD had good diagnostic accuracy for infection presence in both alcohol and HBV-related ACLF patients, especially in HBV-related ACLF patients with AUROC 0.868. Additionally, WBD also had a higher diagnostic accuracy for infection presence in both early-stage and late-stage ACLF patients, especially in early-stage patients with AUROC 0.873.

This indicated that WBD holds the potential for assessing the probability of infection across varying etiologies and stages of ACLF. Simultaneously there is an encouragement for conducting larger-scale multicenter studies encompassing patient cohorts with diverse etiologies and stages.

## Methods

### Participants

A total of 185 participants were retrospectively enrolled in this study, comprising 125 individuals from Shandong Provincial Hospital Affiliated to Shandong First Medical University (May 2013 to July 2021) and 60 from Jinan Center Hospital of Shandong First Medical University (October 2011 to April 2022) (Table. S2). The research protocol was approved by the Ethics Committee of Shandong Provincial Hospital Affiliated with Shandong First Medical University and Jinan Central Hospital Affiliated with Shandong First Medical University. All methods were performed by the relevant guidelines and regulations. Written informed consent was obtained from all participants. Exclusion criteria were as follows: **(**1) younger than 18 years. or older than 80 years.; (2) infection was clearly diagnosed before hospitalization; (3) having a history of hepatocellular carcinoma or other malignancies; (4) receiving immunosuppressive drugs for reasons other than chronic liver disease; (5) insufficient medical records. The diagnostic criteria of ACLF were based on the 2018 Guidelines for the diagnosis and treatment of liver failure in China from Chinese Medical Association (CMA)^1^. Infection was diagnosed through a combination of clinical features, laboratory tests, and imaging findings. The criteria for the diagnosis of bacterial infection were as follows^2^: (1) Spontaneous bacterial peritonitis (SBP): polymorphonuclear cell count in ascitic fluid ≥ 250/mm^3^; (2) pulmonary infection: clinical signs of bacterial infection and new infiltrates on chest computed tomography (CT), and clinical features of infection, no radiographic infiltrates, and positive sputum culture; (3) Urinary tract infection (UTI): abnormal urinary sediment (> 10 leukocytes/field) and positive urinary culture or uncountable leukocytes per field there was a negative urinary culture; (4) Skin and soft tissue infections (SSTIs): clinical signs of infection associated with swelling, erythema, heat, and tenderness in the skin; (5) Spontaneous bacteremia (SB): positive blood cultures and no cause of bacteremia. (6) Fungal infection (FI)^44^: isolation of fungal in one or more blood cultures, or detection of fungal by direct examination and/or culture of respiratory samples in the presence of radiological imaging compatible with the lung infection.

### Etiology and stage of ACLF

Patients were classified into HBV-related and non-HBV-related ACLF based on the cause of their chronic liver disease. ACLF stages were divided into prophase, early, middle, and end stages, defined as follows: (1) prophase stage: 40% < prothrombin activity (PTA) < 50% (International normalized ratio (INR) < 1.5); (2) early stage: 30% < PTA < 40% (or 1.5 ≤ INR < 1.9), without complications or extra-hepatic organ failure; (3) middle stage: 20% < PTA < 30% (or 1.9 ≤ INR < 2.6), with one complication and/or one extra-hepatic organ failure; (4) end-stage: PTA < 20% (or INR ≥ 2.6) with two complications and/or two extra-hepatic organ failures^1^.

### Study design

**Figure 6:**
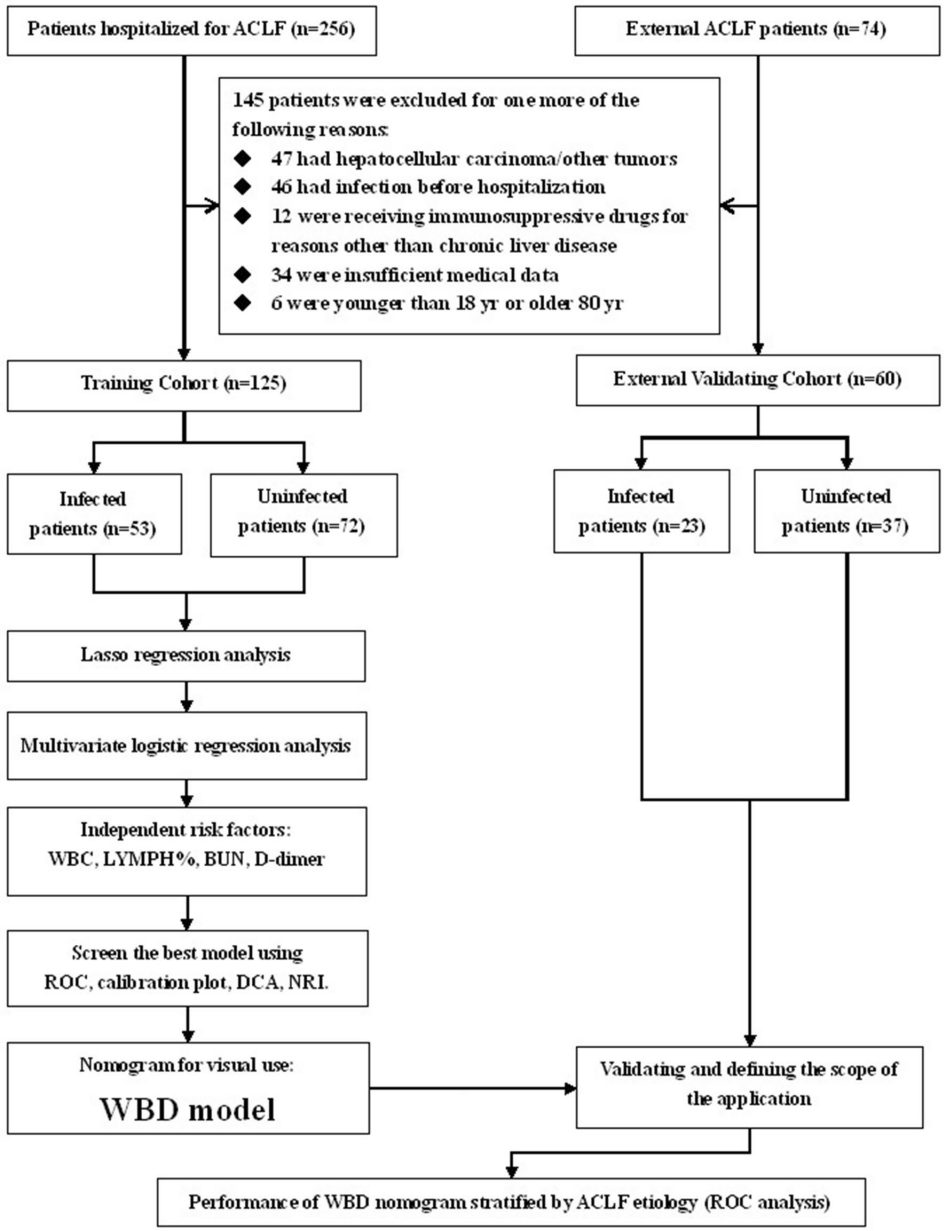
Flow chart of participants’ enrollment, grouping, and the study design.

This study employed a retrospective study design, which ultimately enrolled 125 patients in the training cohort. 60 patients, which were classified as the external validation cohort, were ultimately used to further evaluate the model diagnostic accuracy (Fig. 6). Infection was confirmed by the infection criteria after hospital admission, and patients were categorized as infected and uninfected. The laboratory data, including WBC, RBC, PLT, HGB, NEU%, LYM%, MON%, NEU, LYM, MON, CRP, PCT, ALT, AST, GGT, ALP, ALB, TBIL, DBIL, IBIL, BUN, CRE, PT, INR and D-dimer, were collected at the time of diagnosis of infection presence or with short interval.

### Statistical analysis

Continuous variables were expressed as Mean ± standard deviation or media ± interquartile range using the *t*-test or rank-sum test to determine the statistical significance between the infected and non-infected groups. Categorical variables were indicated as numbers (proportions) and assessed by the Chi-square test for comparing the statistical significance between these two groups. The analysis of collinearity was primarily performed on all variables between these two groups in the training cohort. All selected variables were analyzed by lasso regression analysis to confirm the risk factors. Multivariate logistic analysis was used to assess the independent risk factors and facilitated the construction of a nomogram. AUROC to evaluate the diagnostic accuracy. Calibration plots, clinical impact curves, and DCA were performed to further verify the consistency and clinical utility of these models. NRI between these models was calculated to identify the optimized model. Finally, the application of fivefold cross-validation divided the entire dataset equally into five cross-validation splits. Stratification analysis was performed by the ACLF etiology and stage. R software version 4.1.1 (http://www.rproject.org/) was used for statistics, with* p* < 0.05 considered significant.

### Supplementary Information

Supplementary Legends.

Supplementary Figure S1.

Supplementary Figure S2.

Supplementary Figure S3.

Supplementary Figure S4.

Supplementary Table S1.

Supplementary Table S2.

## Data Availability

The datasets generated and analyzed during the current study are available from the corresponding authors on reasonable request.
