# Supplementary material for: A novel laboratory-based nomogram for assessing infection presence risk in acute-on-chronic liver failure patients
Source: Sci Rep. 2023 Oct 8;13:16970. doi: 10.1038/s41598-023-44006-9 (PMC10560663; doi:10.1038/s41598-023-44006-9)
Supplement: Supplementary file 1 — Supplementary Legends. [file 41598_2023_44006_MOESM1_ESM.docx]

**Fig.S1 The distribution of the independent variables with significant differences between the infected and non-infected patients in the training cohort**

The scatter plot of WBC count (a), LYM% (b), NEU% (c), MON count (d), NEU count (e), ALP (f), IBIL (g), BUN (h), CRE (i), D-dimer (j) were plotted to compare the distribution of these variables between ACLF patients with/without infection. Note: ^*^*p* < 0.05, ^**^ *p* < 0.01, ^***^ *p* < 0.001 for significance

**Fig.S2 Multicollinearity Heatmap of the independent variable and Variable selection by LASSO logistic regression in the Training cohort**

The Multicollinearity Heatmap of independent variables in the training cohort (a); the LASSO coefficient profile for 11 risk factors (b). Cross-validation curves (c).

**Fig.S3 Correlation between WBC count, BUN, and D-dimer**

The Correlation between WBC count and BUN(a), WBC count and D-dimer(b), and BUN and D-dimer(c) in the training cohort.

Note: Correlation coefficient: R<0.3 No correlation; 0.3<R<0.8 weak correlation; R>0.8 strong correlation; ^**^*p*<0.001

**Fig.S4** **Receiver operating characteristic curve (ROC) analysis of WBD in training cohort by the fivefold cross-validation**

ROC curves of WBD in the first training cohort (a), the second training cohort (b), the third training cohort (c), the fourth training cohort, and (d), the fifth training cohort (e).
