# Supplementary figures and images for: A novel laboratory-based nomogram for assessing infection presence risk in acute-on-chronic liver failure patients
Source: Sci Rep. 2023 Oct 8;13:16970. doi: 10.1038/s41598-023-44006-9 (PMC10560663; doi:10.1038/s41598-023-44006-9)

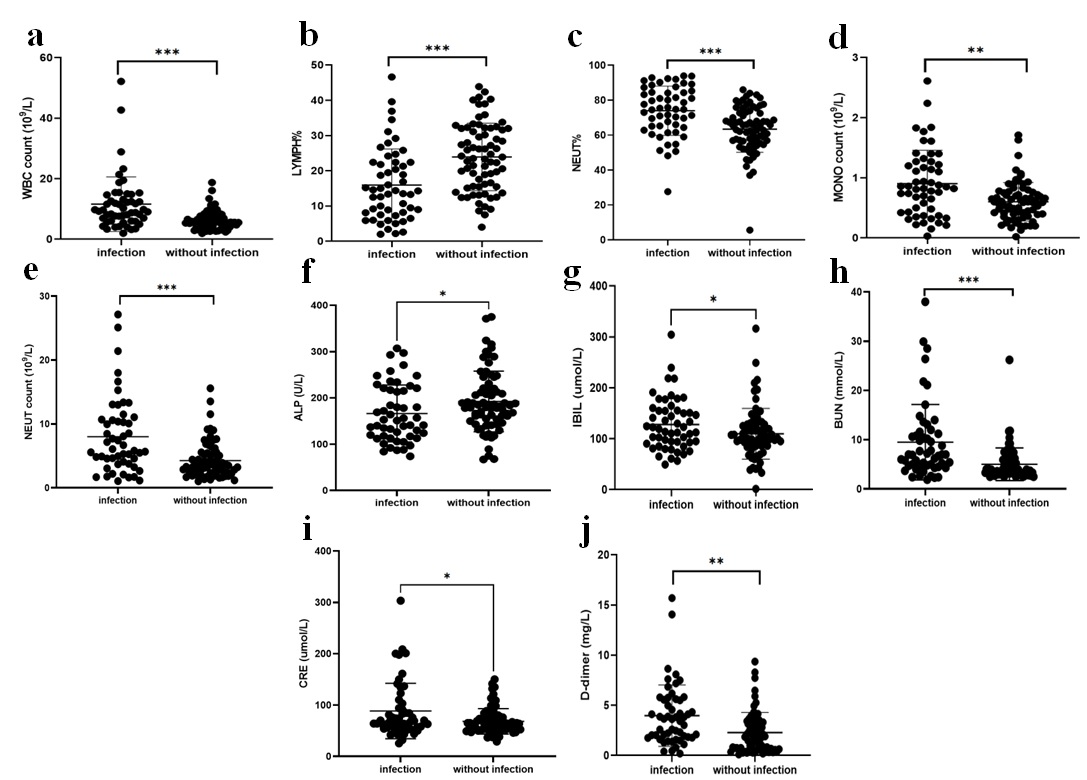

Supplement: Supplementary file 2 — Supplementary Figure S1. [file 41598_2023_44006_MOESM2_ESM.jpg]

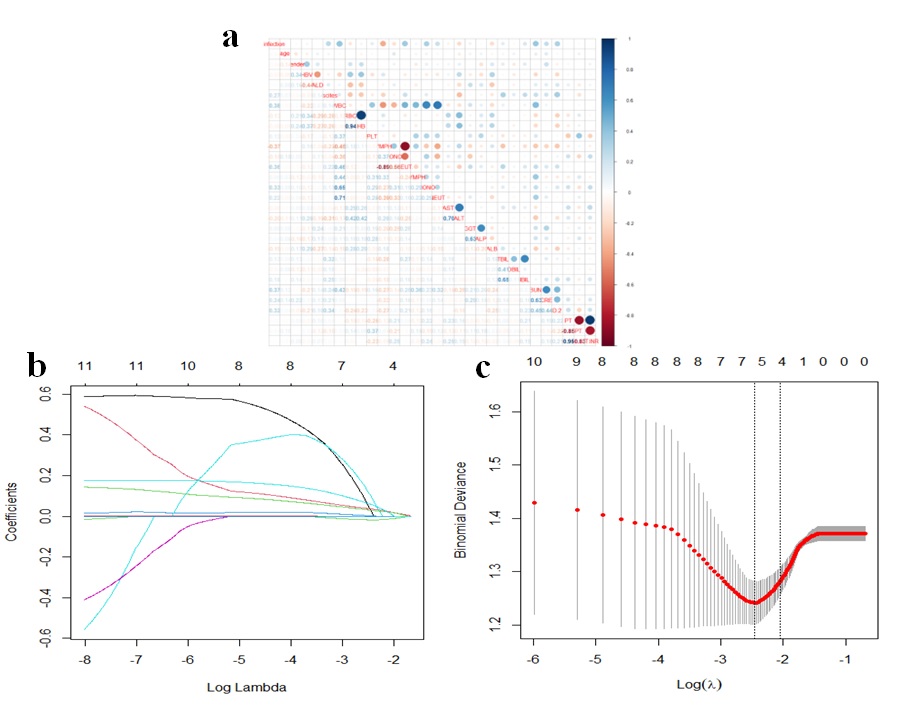

Supplement: Supplementary file 3 — Supplementary Figure S2. [file 41598_2023_44006_MOESM3_ESM.jpg]

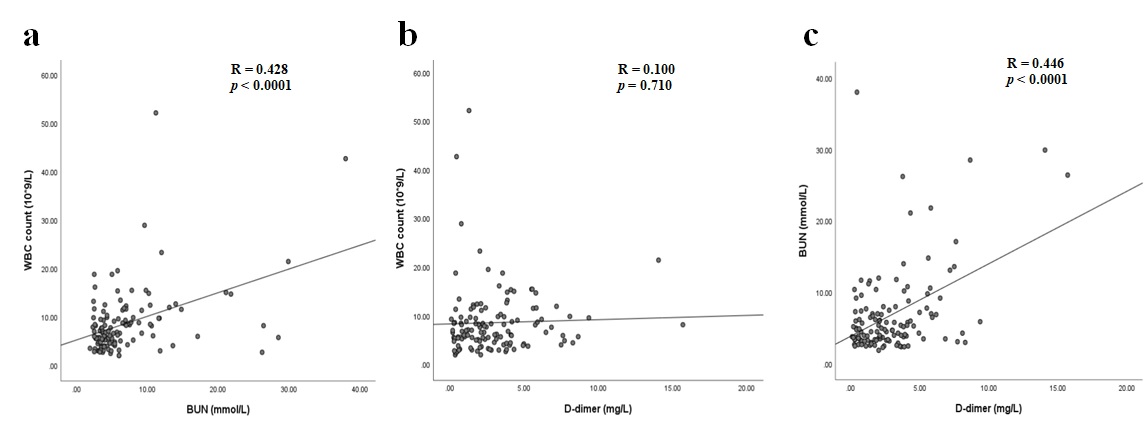

Supplement: Supplementary file 4 — Supplementary Figure S3. [file 41598_2023_44006_MOESM4_ESM.jpg]

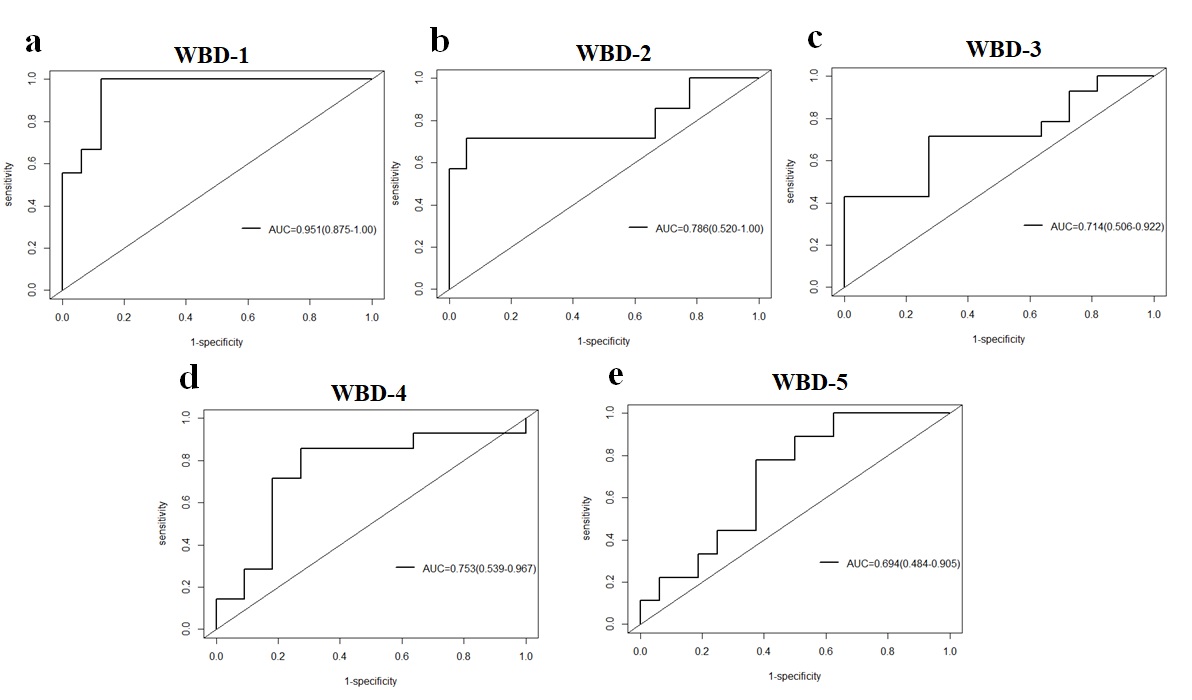

Supplement: Supplementary file 5 — Supplementary Figure S4. [file 41598_2023_44006_MOESM5_ESM.jpg]
