# Supplementary material for: A novel laboratory-based nomogram for assessing infection presence risk in acute-on-chronic liver failure patients
Source: Sci Rep. 2023 Oct 8;13:16970. doi: 10.1038/s41598-023-44006-9 (PMC10560663; doi:10.1038/s41598-023-44006-9)
Supplement: Supplementary file 6 — Supplementary Table S1. [file 41598_2023_44006_MOESM6_ESM.docx]

| Multicollinearity test | | | |
| --- | --- | --- | --- |
| Variables（n=26） | Tolerance | | Variance inflation factor |
| Age (years) | 0.780 | 1.282 | |
| Gender | 0.545 | 1.835 | |
| Ascites | 0.551 | 1.814 | |
| WBC (10^9^/L) | 0.042^*^ | 24.012^**^ | |
| RBC (10^12^/L) | 0.085^*^ | 11.744^**^ | |
| HB (g/L) | 0.078^*^ | 12.883^**^ | |
| PLT (10^9^/L) | 0.451 | 2.217 | |
| LYM% | 0.030^*^ | 33.142^**^ | |
| MON% | 0.146 | 6.861^*^ | |
| NEU% | 0.021^*^ | 48.317^**^ | |
| LYM (10^9^/L) | 0.248 | 4.038 | |
| MON (10^9^/L) | 0.144 | 6.931^*^ | |
| NEU (10^9^/L) | 0.056^*^ | 17.729^**^ | |
| AST(U/L) | 0.380 | 2.629 | |
| ALT(U/L) | 0.327 | 3.060 | |
| GGT(U/L) | 0.447 | 2.237 | |
| ALP (U/L) | 0.412 | 2.428 | |
| ALB (g/L) | 0.686 | 1.459 | |
| TBIL (μmol /L) | 0.334 | 2.994 | |
| DBIL (μmol /L) | 0.637 | 1.570 | |
| IBIL (μmol /L) | 0.439 | 2.275 | |
| BUN (mmol/L) | 0.218 | 4.582 | |
| CRE (μmol /L) | 0.289 | 3.456 | |
| D-Dimer (mg/L) | 0.478 | 2.091 | |
| PT(s) | 0.061^*^ | 16.409^**^ | |
| INR | 0.183 | 5.460^*^ | |

Table.S1 Multicollinearity test between independent variables in the training cohort

Note: Existence of Multicollinearity: ^*^Tolerance< 0.1; ^**^Variance inflation factor> 10; ^*^Variance inflation factor> 5.
