# Supplementary material for: A novel laboratory-based nomogram for assessing infection presence risk in acute-on-chronic liver failure patients
Source: Sci Rep. 2023 Oct 8;13:16970. doi: 10.1038/s41598-023-44006-9 (PMC10560663; doi:10.1038/s41598-023-44006-9)
Supplement: Supplementary file 7 — Supplementary Table S2. [file 41598_2023_44006_MOESM7_ESM.docx]

Table. S2 Baseline characteristics of ACLF patients in the external validating cohort and comparison with those of the training cohort

| Variables | Training Cohort | External Validating Cohort | | *p* |
| --- | --- | --- | --- | --- |
| (n = 32) | (n =125) | (n = 60) | |  |
| Age (IQR) (years) | 48.00 (41.00, 55.00) | 46.00 (40.00, 51.25) | 0.339 | |
| Infection, n (%) |  |  |  | |
| present | 53 (42.4) | 23 ( 38.3) | 0.635 | |
| absent | 72 (57.6) | 37 ( 61.7) |  | |
| SBP, n (%) | 23 (43.4) | 11(47.8) |  | |
| Pneumonia, n (%) | 17 (32.1) | 12 (52.2) |  | |
| UTI, n (%) | 6 (11.3) | - |  | |
| SSTIs, n (%) | 3 (5.7) | - |  | |
| SB, n (%) | 2 (3.8) | - |  | |
| FI, n (%) | 5 (9.4)^§^ | - |  | |
| Gender, n (%) |  |  |  | |
| Male | 99 (79.2) | 48 ( 80.0) | 1 | |
| Female | 26 (20.8) | 12 ( 20.0) |  | |
| Alcohol abuse, n (%) |  |  |  | |
| present | 37 (29.6) | 11 ( 18.3) | 0.111 | |
| absent | 88 (70.4) | 49 ( 81.7) |  | |
| HBV infection, n (%) |  |  |  | |
| present | 94 (75.2) | 51 ( 85.0) | 0.181 | |
| absent | 31 (24.8) | 9 ( 15.0) |  | |
| Ascites, n (%) |  |  |  | |
| present | 97 (77.6) | 45 ( 75.0) | 0.713 | |
| absent | 28 (22.4) | 15 ( 25.0) |  | |
| WBC (IQR) (10^9^/L) | 6.77 (4.75, 9.81) | 7.66 (6.12, 9.70) | 0.107 | |
| RBC (IQR) (10^12^/L) | 3.89 (3.36, 4.43) | 4.20 (3.43, 4.56) | 0.121 | |
| HB (IQR)(g/L) | 125.00 (109.00, 141.00) | 131.00 (118.75, 143.00) | 0.23 | |
| PLT (IQR)(10^9^/L) | 90.00 (63.00, 141.00) | 108.50 (76.25, 143.75) | 0.233 | |
| LYM% (IQR) | 19.90 (12.50, 28.00) | 15.20 (9.55, 20.52) | 0.003 | |
| MON% (SD) | 9.20 (6.90, 11.80) | 8.55 (6.75, 10.77) | 0.451 | |
| NEU% (IQR) | 67.70 (58.80, 78.70) | 74.05 (66.55, 81.05) | 0.009* | |
| LYM (IQR)(10^9^/L) | 1.21 (0.89, 1.65) | 1.15 (0.85, 1.47) | 0.413 | |
| MON (IQR)(10^9^/L) | 0.66 (0.40, 0.90) | 0.72 (0.46, 1.06) | 0.105 | |
| NEU (IQR)(10^9^/L) | 4.42 (2.64, 7.63) | 5.64 (3.67, 7.22) | 0.181 | |
| PCT (IQR)(ng/ml) | 0.56 (0.40, 0.72) | 0.57 (0.44, 0.71) | 0.913 | |
| CRP (IQR)(ng/ml) | 11.32 (7.46, 18.83) | 11.82 (6.25, 19.93) | 0.901 | |
| AST (IQR)(U/L) | 161.00 (85.00, 383.00) | 143.50 (103.00, 240.50) | 0.692 | |
| ALT (IQR)(U/L) | 153.00 (58.00, 394.00) | 162.50 (66.00, 358.25) | 0.772 | |
| GGT (IQR)(U/L) | 98.00 (56.00, 148.00) | 94.50 (50.75, 136.48) | 0.621 | |
| ALP (IQR)(U/L) | 178.00 (135.00, 221.00) | 146.50 (116.75, 176.75) | 0.001* | |
| ALB (IQR)(g/L) | 29.40 (27.00, 33.30) | 32.45 (29.87, 35.52) | 0.001* | |

**p* < 0.05 for significance；^§^ Among them, two individuals had pulmonary fungal infections, and one had a cutaneous fungal infection.

Table. S2 (Continued)

Baseline characteristics of ACLF patients in the external validating cohort and comparison with those of the training cohort

| Variables | Training Cohort | External Validating Cohort | *p* |
| --- | --- | --- | --- |
| (n = 32) | (n =125) | (n = 60) |  |
| TBIL (IQR)(μmol /L) | 198.00 (114.00, 287.03) | 211.00 (171.28, 275.78) | 0.198 |
| DBIL (IQR)(μmol /L) | 109.30 (86.10, 141.20) | 107.00 (83.57, 148.48) | 0.908 |
| IBIL (IQR)(μmol l/L) | 319.70 (211.40, 412.10) | 310.00 (261.52, 429.68) | 0.327 |
| BUN (IQR)(mmol/L) | 4.90 (3.50, 7.50) | 4.24 (3.29, 5.83) | 0.064 |
| CRE (IQR)(μmol L) | 64.00 (53.00, 82.94) | 59.50 (53.00, 67.25) | 0.094 |
| D-dimer (IQR)(mg/L) | 2.30 (1.10, 4.03) | 2.12 (0.71, 3.33) | 0.184 |
| PT (IQR)(s) | 21.80 (18.10, 26.80) | 24.15 (21.05, 30.10) | 0.002* |
| INR (IQR) | 1.90 (1.56, 2.37) | 2.24 (1.89, 2.63) | <0.001* |

**p* < 0.05 for significance；
